# Integrative Transcriptomics and Proteomics Analysis Provide a Deep Insight Into Bovine Viral Diarrhea Virus-Host Interactions During BVDV Infection

**DOI:** 10.3389/fimmu.2022.862828

**Published:** 2022-03-16

**Authors:** Yingying Ma, Li Wang, Xiaoxia Jiang, Xin Yao, Xinning Huang, Kun Zhou, Yaqi Yang, Yixin Wang, Xiaobo Sun, Xueting Guan, Yigang Xu

**Affiliations:** ^1^ College of Veterinary Medicine, Northeast Agricultural University, Harbin, China; ^2^ College of Animal Science and Technology, Northeast Agricultural University, Harbin, China; ^3^ Key Laboratory of Applied Technology on Green-Eco-Healthy Animal Husbandry of Zhejiang Province, College of Animal Science and Technology, College of Veterinary Medicine, Zhejiang A&F University, Hangzhou, China; ^4^ Zhejiang Provincial Engineering Research Center for Animal Health Diagnostics and Advanced Technology, College of Animal Science and Technology, College of Veterinary Medicine, Zhejiang A&F University, Hangzhou, China

**Keywords:** BVDV, BVDV-host interaction, transcriptomics, proteomics, integrative analysis

## Abstract

Bovine viral diarrhea virus (BVDV) is the causative agent of bovine viral diarrhea-mucosal disease (BVD-MD), an important viral disease in cattle that is responsible for extensive economic losses to the cattle industry worldwide. Currently, several underlying mechanisms involved in viral replication, pathogenesis, and evading host innate immunity of BVDV remain to be elucidated, particularly during the early stage of virus infection. To further explore the mechanisms of BVDV-host interactions, the transcriptomics and proteomics profiles of BVDV-infected MDBK cells were sequenced using RNA-seq and iTRAQ techniques, respectively, and followed by an integrative analysis. Compared with mock-infected MDBK cells, a total of 665 differentially expressed genes (DEGs) (391 down-regulated, 274 up-regulated) and 725 differentially expressed proteins (DEPs) (461 down-regulated, 264 up-regulated) were identified. Among these, several DEGs and DEPs were further verified using quantitative RT-PCR and western blot. Following gene ontology (GO) annotation and KEGG enrichment analysis, we determined that these DEGs and DEPs were significantly enriched in multiple important cellular signaling pathways including NOD-like receptor, Toll-like receptor, TNF, NF-κB, MAPK, cAMP, lysosome, protein processing in endoplasmic reticulum, lipid metabolism, and apoptosis signaling pathways. Significantly, the down-regulated DEGs and DEPs were predominantly associated with apoptosis-regulated elements, inflammatory factors, and antiviral elements that were involved in innate immunity, thus, indicating that BVDV could inhibit apoptosis and the expression of host antiviral genes to facilitate viral replication. Meanwhile, up-regulated DEGs and DEPs were primarily involved in metabolism and autophagy signaling pathways, indicating that BVDV could utilize the host metabolic resources and cell autophagy to promote replication. However, the potential mechanisms BVDV-host interactions required further experimental validation. Our data provide an overview of changes in transcriptomics and proteomics profiles of BVDV-infected MDBK cells, thus, providing an important basis for further exploring the mechanisms of BVDV-host interactions.

## Introduction

Bovine viral diarrhea virus (BVDV), a member of the genus *Pestivirus* of the *Flaviviridae*, is the causative agent of bovine viral diarrhea-mucosal disease (BVD-MD) that results in diarrhea and mucosal erosions accompanied by respiratory and reproductive diseases (1). BVD-MD is an important disease in cattle and can severely threaten the development of animal husbandry, causing high economic losses to the cattle industry worldwide (2). Furthermore, BVDV-susceptible animals also include goats, sheep, camels, and pigs (3–5). BVDV with envelope is a spherical particle 40-60 nm in diameter, which contains a positive-sense single-stranded RNA genome that is approximately 12.3 kb in length (6). Currently, BVDVs are divided into two major genotypes: BVDV-1 and BVDV-2, which comprise multiple subgenotypes based on their genetic heterogeneity (7, 8). Additionally, according to the cytopathic effect (CPE) of BVDV-infected cells, BVDV is divided into cytopathic (CP) and non-cytopathic (NCP) types. The NCP BVDV often leads to persistent infection (PI) and immunosuppression in cattle (9). Throughout their entire life cycle, PI cattle can continue to spread the virus to other animals, and this is considered to be the primary source of BVDV transmission in cattle farms (9). So, it is of great importance to eliminate PI cattle to effectively control BVD-MD outbreaks in cattle. To date, vaccination remains the primary means of preventing BVDV infection.

Typically, the viral life cycle primarily consists of four stages including adsorption, replication, assembly, and release, and this process is quite complex. BVDV begins its life cycle from endocytosis through the binding of the viral membrane protein to the host cell surface receptor (10). The BVDV envelope proteins E1, E2, and Erns play important roles during this process (11). The BVDV E2 protein has been confirmed to bind to low-density lipoprotein (LDL) on the surface of host cells, thus, mediating BVDV entry into the target cells (12). However, there are numerous BVDV receptors on the surface of host cells, and these require further elucidation. It has been reported that CD46 may function as one of the cellular receptors of BVDV (13). After viral entry, a large number of positive strand RNA of BVDV are generated under the action of viral proteins possessing enzymatic activity. During this process, BVDV will have to utilize (promote/inhibit) certain host proteins to ensure normal viral replication and progeny virus assembly. Studies have demonstrated that the protease activity of the carboxyl-terminal region of the BVDV non-structural protein NS3 plays an important role in the early replication of BVDV, and the BVDV NS5B protein can also affect early viral replication (14). During the process of virus genomic RNA packaging and release of virus particles, the capsid protein binds to viral genomic RNA in the endoplasmic reticulum cavity and BVDV virions appear to mature in intracellular vesicles at the endoplasmic reticulum or Golgi apparatus (15, 16). However, the mechanisms by which BVDV utilizes host cells to complete viral replication and virions release are still unknown. Moreover, the underlying mechanisms of immunosuppression, viral pathogenesis, evasion of host innate immunity, and inflammatory reaction caused by BVDV remain unclear.

In recent years, the differential transcriptomics and proteomics data before and after viral infection has allowed us to systematically analyze the virus-infected host to determine potential biomarkers that play key roles in virus-host interactions (17, 18). For example, Xin *et al.* performed quantitative proteomic analyses of Zika virus (ZIKV)-infected C6/36 cells, and identified 200 differentially expressed proteins (DEPs), and based on the differential proteomics, they further found that the host protein CHCHD2 played a key role in promoting the replication of ZIKV and inhibiting the production of IFN-β (19). Using RNA-seq-based transcriptome analysis for PEDV-infected Vero cells, researchers observed that significant changes in the mTOR signaling pathway occurred at different time points after viral infection, and this was further experimentally confirmed to promote virus infection (20). Moreover, there have been several studies that report transcriptomic changes (21) and proteomic changes (22) in BVDV-infected host cells. However, only one omics data type analysis is limited to correlation between mRNA and protein abundances, such as transcriptomic data only represents the mRNA expression level, but not represents the presence of post-translational modifications, which was insufficient to predict protein expression levels from quantitative mRNA data. Undoubtedly, integrated analysis of multi-omics data would provide a comprehensive view of the underlying mechanisms of pathogen (virus/bacterium/parasite)-host and microbe-environmental interactions (23–26). 

To date, the underlying mechanisms of pathogenesis, viral replication, and evasion of host innate immunity during the process of BVDV infection remain to be elucidated, particularly during the early stage of virus infection. Thus, to further explore the mechanisms of BVDV-host interactions, we obtained RNA-seq-based transcriptomics profiles and iTRAQ-based proteomics profiles of BVDV-infected MDBK cells were obtained and further conducted an integrative analysis.

## Materials and Methods

### Virus and Cell

Madin-Darby bovine kidney (MDBK) cells were cultured in Dulbecco’s Modified Eagle Medium (DMEM) (Gibco, USA) supplemented with 10% fetal bovine serum (Gibco, USA) at 37°C in a 5% CO_2_ incubator. The BVDV strain VEDEVAC AV69 from our laboratory was propagated in MDBK cells, followed by the CPE observation; identification by an indirect immunofluorescence assay (IFA) using mouse anti-E2 monoclonal antibody (1:500) prepared in our laboratory as the primary antibody and fluorescein isothiocyanate (FITC)-labeled goat anti-mouse IgG antibody (1:1000) (Thermo Fisher Scientific, USA) as the secondary antibody; and determination of the one-step growth curve of BVDV with a viral titer that was expressed as the 50% tissue culture infective dose (TCID_50_). MDBK cells (2×10^6^/mL) were seeded into 6-well cell culture plates and cultured for 24 h in a 5% CO_2_ incubator. Next, the MDBK cells were inoculated with BVDV at a multiplicity of infection (MOI) of 1.0 and cultured for 48 h and used as the experimental group (BVDV-infected MDBK group). MDBK cells cultured in DMEM were used as the control group (mock-infected MDBK group).

### RNA Extraction, Sequencing, and Bioinformatics Analysis

Total RNA from the BVDV-infected MDBK and the mock-infected MDBK cells was extracted (three biological replicates per group) using TRIzol reagent (Invitrogen, USA) according to the manufacturer’s instructions. The purity, concentration, and integrity of RNA were assessed using an Agilent 2100 Bioanalyzer (Agilent Technologies, USA). After removing ribosomal RNAs (rRNAs) from the total RNA extractions using a Ribo-zero TM rRNA Removal Kit (Epicentre, USA), messenger RNAs (mRNAs) were enriched and fragmented using fragmentation buffer and then reverse transcribed into cDNA using random primers (Thermo Fisher Scientific, USA). This was followed by second-strand cDNA synthesis, and the cDNA fragments were purified using a QIAquick Gel Extraction Kit (Qiagen, Germany). These fragments were then end repaired, treated with poly(A), and ligated to Illumina sequencing adapters. Subsequently, the ligation products were size-selected according to agarose gel electrophoresis, followed by PCR amplification and high-throughput sequencing on an Illumina HiSeqTM 4000 platform. After sequencing, high-quality reads (clean reads) were obtained, by removing low-quality reads and rRNA-mapped reads, and mapped to the reference genome using TopHat (v2.0.9). The gene expression level was normalized using the FPKM (fragments per kilobase of transcript per million mapped reads) method, and the genes with a |log2FC| > 1 and a false discovery rate (FDR) < 0.05 were identified as significantly differentially expressed genes (DEGs). The DEGs were then subjected to gene ontology (GO) database analysis for functional annotation and Kyoto Encyclopedia of Genes and Genomes (KEGG) database analysis to identify the enriched pathways they were involved in. GO enrichment analysis provides all GO terms that are significantly enriched in DEGs in comparison to the genome background and then filters the DEGs that correspond to biological functions. KEGG is a database resource for the systematic analysis of gene functions in terms of the networks of genes and molecules, and significantly enriched metabolic pathways or signal transduction pathways of the DEGs in comparison to the whole genome background. All the raw data were deposited into the NCBI database as a BioProject (accession no. PRJNA596327).

### Validation of RNA-Seq Results by RT-qPCR

To verify the reliability of RNA-seq sequencing data, quantitative RT-PCR (RT-qPCR) was employed to validate the DEGs that were identified from transcriptome sequencing. There were 10 DEGs that were randomly selected from the RNA-seq sequencing data: CACNA1S, GFAP, Grin2a, NR4A1, DHCR24, NOD2, IL6, IL2RB, BCL2A1, and CCR8. Moreover, a number of key molecular elements were involved in important signaling pathways, including apoptosis signaling pathway, RIG-I-like receptor signaling pathway, NF-κB signaling pathway, mTOR signaling pathway, Toll-like receptor signaling pathway, AMPK signaling pathway, protein processing in endoplasmic reticulum pathway, and metabolic pathway. BVDV-infected MDBK cell samples were prepared as described above, and mock-infected MDBK cell samples were used as a control. The extracted total RNA was subjected to SYBR Green-based RT-qPCR, using Roche SYBR qPCR Master Mix (Roche, USA) and the primers (**Table 1**), on an ABI 7500 system (Applied Biosystem, USA), and β-actin gene was used as an internal reference gene. Relative expression levels of the target genes were calculated using the 2^-ΔΔCt^ method. All RT-qPCRs were performed in three technical replicates.

**Table 1:** Primers used in this study.

Genes	Primer sequences (5’-3’)	Accession number	Genes	Primer sequences (5’-3’)	Accession number
β-actin	F:GCCAACCGTGAGAAGATGAC	AY141970.1	FASN	F: ACCTGCACTTCCACAACCCAAAC	NM_001012669.1
	R:AGGCATACAGGGACAGCACA			R: ACCGAAGCCAAAGGAGTTGATGC	
GFAP	F:CTGCGGCTCGATCAACTCACTG	XM_010816312.3	DHCR7	F: ACATGCTGCTTCCTGACTTCTGC	NM_001014927.1
	R:GGTGGCTTCATCTGCTTCCTGTC			R: ATCTCATACTTGTTGACGGCTCCTG	
NOD2	F:CGCACGGAACTCAGCCTCAAG	NM_001002889.1	SCD1	F: TATCCGACCTAAGAGCCGAGAAGC	NM_173959.4
	R:ATCCAGGAGAAGACAGGCAGGTG			R: TGGGCAGGATGAAGCACAACAAC	
CCR8	F:CTCGCTCCTCCACCGTAGACTAC	NM_001194962.1	HMGCR	F: AGCCATTTTGCCCGAGTCTTAGAAG	NM_001105613.1
	R:GCCTCCAACTCCAGTGTGAATCG			R: AGCGTGAACAAGAACCAAGCCTAG	
IL2RB	F:ATTGACGGTGATGTGCTCTGAAGG	NC_037332.1	TNFα	F: GCTGACGGGCTTTACCTCATCTAC	NM_173966.3
	R:TGTGGACGACCTGGAGTGAGTG			R: GGCTCTTGATGGCAGACAGGATG	
BCL2A1	F:AAGCGACCAGGAGGAAGGATAGC	NM_001114735.2	IL12	F: CTGTGGTGAGAAGTTGACTCTGGAC	HS091876.1
	R:GCAAGTGACCGAGACAGTCTGAAG			R: GGACATGCCTTGCTGGTTGGAG	
DHCR24	F:CGCGTGCGGGACATCCAGAA	NC_037330.1	JUN	F: GAACTCCGACCTCCTCACCTCTC	XM_027537105.1
	R:GCTCCACTCGGACAATCTGT			R: CCCGTTGCTGGACTGTATGATTAGG	
NR4A1	F:AGCCGTACACCTGGAAGTCCTC	NM_001075911.1	MAP3K7	F: CCGCTTCTTCTTCTTCCTCGTCTTC	NM_001081595.1
	R:GTGCTCCTCTGCCTCCTCCTC			R: GCTCCTCTTCCAACAACCTCTTCC	
Grin2a	F:CGTCGGCTCACTCACCATCAAC	XM_024985455.1	IFNα	F: AAGTGTTGCGTTTAGCGGAGGAC	NM_001029845.3
	R:CGCTGATTCCTGTCTCCACGAAG			R: TTGTGTCTGCCATTGTCTTGAGAGG	
IL6	F:CACTGACCTGCTGGAGAAGATGC	NM_173923.2	IRF7	F: TTGACTTCGGCACCTTCTTCCAAG	NM_001105040.1
	R:CCGAATAGCTCTCAGGCTGAACTG			R: TTCACCAGGACCAGGCTCTTCTC	
CACNA1S	F:AGCACCACCACCAGCCTCTC	XM_024976575.1	MAVS	F: TGGCAGGCTGGTATCTAGGATGG	NM_001046620.2
	R:AGCAGTCGAAGCGGTTGAAGATG			R: CAAGGAGTTACTGTGGCTGATGGC	
AMPK	F:ACAGCCGAGAAGCAGAAACACG	NM_001109802.2	RIG-I	F: CGTGGCAGAACAAATCAGACAATGG	XM_027549061.1
	R:TCAGGAAGAGCAAGAGAAGGAAGGG			R: GGCGACCGAGGTAGCAATTAGAATC	
GRP94	CGCAGGAACAGACGAGGAAGAAC	AB025193.1	ERP57	F: GATTGAGTTCTATGCCCCGTGGTG	EG343338.1
	CACATTCCCTCTCCACACAGCATC			R: TCTGATTCAGCGGACAGTTTCTTGG	
SERP2	GAAGCCGACAAGTCACCCAGAAG	NM_001113721.1	ULK1	F: GAGCATCGGCACCATCGTGTAC	NM_001002889.1
	GTACCGAGATGTCCTGTGAGAAAGC			R: GACCAGCGTCTTGTTCTTCTCGTAG	
CRT	TCTGGCACCATCTTTGACAACTTCC	AB067687.1	Beclin-1	F: ACTGGACACGAGCTTCAAGATTCTG	NM_001033627.2
	CTCCTCCTCCTCATGTAGCCTCTG			R: CCTCCTGGGTCTCTCCTGGTTTC	
UGGT	GCTGTGCTGCATTTGTGGTTATCC	NM_079427.3	ATG5	F: AGCATCATCCCGCAACCAACAG	NM_001034579.2
	AGATACGGTCTGGGCCTTGAAGTAG			R: TGCCTCCACCAAACCTGATTGAAG	
mTOR	CCATCTCGGCAACTTGACCATCC	XM_027564914.1	Caspase-8	F: GTAAGAACCAGCCTCAGCAATCCG	DQ319070.1
	AAGTGCTGCATGTGCTGGAAGG			R: CCTTCACAGCAGCAGCCACTTC	
Caspase-7	AGGAAGCAATGGCAGGGACAAAG	XM_002698509.5	Caspase-3	F: GGTGCCCAGGGAAACTGAAG	NM_001077840.1
	AAGGAAGATTACAGGGCAGGAGGAG			R: TCACGGGAACCAGTGGGTTA	
DDF45	GGTATTGGCTGGGAATGAGAAGTGG	XM_027564945.1	FADD	F: CGGCACCTCGGAGTATCTGACG	XM_027531722.1
	CCCGCTGTCTGTTTCATCTCTGTC			R: GCGTTCTCCCTAGTGCTGTTCTTC	

### Protein Extraction and iTRAQ Labelling

BVDV-infected MDBK cells and mock-infected MDBK cells were prepared as described above. Cells from each group were collected using a cell scraper (three biological replicates per group), and lysed by sonication in a lysis buffer containing 2% SDS, 8 M urea, and complete protease inhibitor cocktail (Roche, USA) at 4°C for 3 min. Then, the cell lysates were centrifuged at 15,000 rpm for 15 min at 4°C, and the supernatant was collected for the determination of total protein concentration using a BCA Protein Assay Kit (Abcam, USA). Subsequently, 100 μg of protein from each sample was digested overnight with sequence-grade modified trypsin (Promega, USA) at 37°C, and the digests were then were dissolved in 500 mM tetraethylammoniumbromide (TEAB) (Sigma, USA). Finally, the resultant peptides were labeled using an iTRAQ 8-plex reagents kit (SCIEX, USA) for 2 h followed by vacuum drying.

### High pH Reverse Phase Separation and LC-MS/MS Analysis

The resultant peptides were re-dissolved in buffer A (20 mM of ammonium formate aqueous solution, pH 10) and then fractionated using the ultimate 3000 system (Thermo Fisher Scientific, USA) that was connected to a reversed-phase chromatographic column. Within 40 minutes, a linear gradient of buffer B (80% of 20 mM ammonium formate plus 20% acetonitrile solution, pH 10) from 5% to 45% was used, followed by a high pH separation (the flow rate was maintained at 1 mL/min, and the temperature was maintained at 30°C), where each component was collected and dried in vacuum. After re-suspension in 30 μL of solvent C (0.1% formic acid aqueous solution), all peptide samples were separated using an online nanoflow EASY-nLC 1000 UHPLC system (Thermo Fisher Scientific, USA) and analyzed by electrospray ionization tandem mass spectrometry (SCIEX, USA). Briefly, 10 μL of peptide was loaded onto a trap column (Thermo Scientific Acclaim PepMap C18, 100 μm × 2 cm) with a flow rate of 10 μL/min for 3 min and then separated using a column (Acclaim PepMap C18, 75 μm × 15 cm) with a linear gradient of acetonitrile solution from 2% to 40% in 70 min (the flow rate was maintained at 300 nL/min). Data were collected by an Orbitrap fusion mass spectrometer that automatically switched between MS and MS/MS, and a full-scan MS spectrum (m/z 350–1550) with a resolution of 120 K was obtained. This was followed by high energy collision dissociation MS/MS scanning at a resolution of 30K. Finally, the strong signals in the MS spectrum (>1e4) were subjected to MS/MS analysis, and the isolation window was 1.6 da.

### Protein Bioinformatics Analysis

Mascot search engine (v2.3.2) was used to analyze the mass spectrometry data in MGF format, followed by the establishment of the database using the reference transcriptomes in NCBI nr/SwissProt/UniProt/IPI databases. Subsequently, the database was compared to the reference genome databases from Macot search engine for the identification of peptide and protein. The proteins with unique spectra ≥ 2 were quantitatively analyzed, and the proteins with *P*-value < 0.05 were characterized as significantly differentially expression proteins (DEP). Next, the DEPs were subjected to GO database analysis and KEGG database analysis for their functional annotation and pathway enrichment, respectively. All raw sequence reads were deposited into the NCBI database as a BioProject (PRJNA596327).

### Validation of iTRAQ Results by Western Blot

The DEPs MOMS1, DHCR24, and CNN2 were selected to verify the iTRAQ results using western blot. Briefly, the cell samples and protein extractions were prepared as described above in this study, and the cell proteins were then separated by 10% SDS-PAGE. The proteins were then transferred onto a polyvinylidene fluoride (PVDF) membrane, blocked with 5% non-fat milk in blocking buffer (20 mmol/L of PBS containing 0.1% Tween 20), and incubated with rabbit anti-MOMS1/DHCR24/CNN2 polyclonal antibody (diluted at 1:500) (LSBio, USA) as the primary antibody and horseradish peroxidase (HRP)-conjugated goat anti-rabbit IgG (diluted at 1:5000) (Abcam, USA) as the secondary antibody. The immunoblot band was visualized using a chemiluminescent substrate (Thermo Fisher Scientific, USA).

### Statistical Analysis

Data of RT-qPCR are presented as mean ± standard error of three replicates per test in a single experiment repeated three times. Comparisons between the two groups were performed using the Student’s t-test. Comparisons of multiple group data were performed using one-way analysis of variance (ANOVA) followed by Tukey’s *post-hoc* test. *P*-values<0.05 were considered to be statistically significant. Statistical analysis was performed on SPSS software version 20.0 (IBM Corp., USA).

## Results

### Virus Propagation and Overview of Multiomics Sequencing

**Figure 1:**
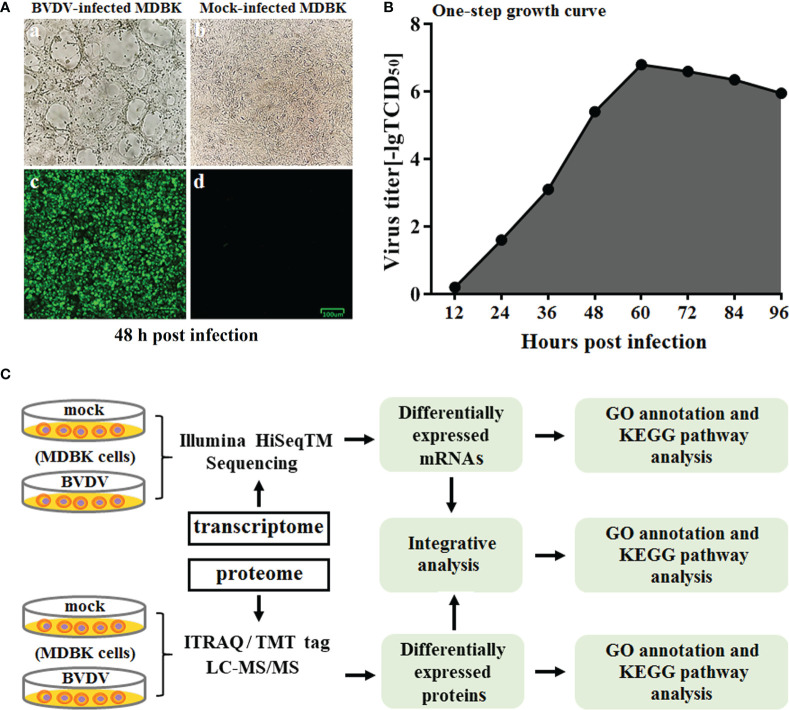
**(A)** Virus identification. a: CPE of BVDV strain AV69; b: mock-infected MDBK cells; c: identification of BVDV by IFA; d: IFA results for mock-infected MDBK cells. **(B)** One-step growth curve of BVDV strain AV69 in MDBK cells. **(C)** Schematic diagram of the transcriptomic and proteomic sequencing workflow.

The BVDV strain used in this study was propagated on MDBK cells followed by CPE observation and IFA identification (**Figure 1A**) at 48 h post BVDV infection. Next, the one-step growth curve characteristics (the replication kinetics curve) of BVDV on MDBK cells were determined. The results revealed that as the virus replicated, virus titers gradually increased. The maximum level of virus titers was observed at 60 h post BVDV infection (**Figure 1B**). Of these, the replication of BVDV was the fastest from 36 to 60 h after viral infection. To further explore the mechanisms of BVDV-host interactions, the BVDV-infected MDBK cell samples were collected at 48 h post infection and subjected to RNA-seq-based transcriptomics and iTRAQ-based proteomics analysis. The overview of multiomics sequencing is presented in **Figure 1C**.

### Quality Evaluation of the Transcriptome and Differentially Expressed Gene Analysis

**Figure 2:**
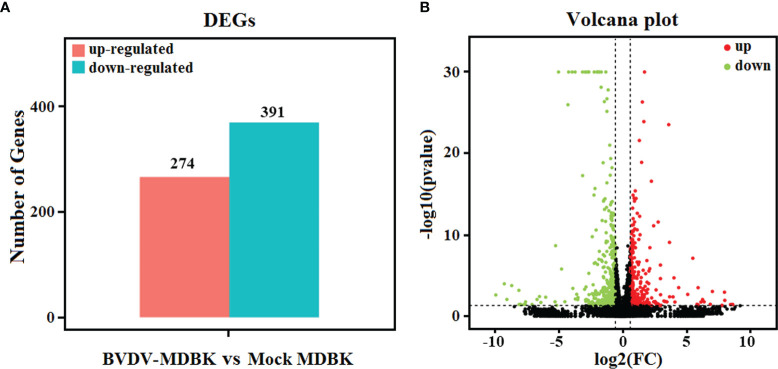
**(A)** Statistics for the DEGs according to the RNA-seq-based transcriptomics data for BVDV-infected MDBK cells compared to mock-infected MDBK cells. The red histogram represents the number of up-regulated DEGs, and the blue histogram represents the number of down-regulated DEGs. **(B)** Volcano plot of the DEGs. The red dots represent significantly up-regulated genes, green dots represent significantly down-regulated genes, and black dots represent insignificant DEGs.

Using mock-infected MDBK cells as a negative control, transcriptome sequencing of the BVDV-infected MDBK cells was performed on an Illumina HiSeqTM 4000 platform. After sequencing, the clean reads from the initial filtering must be re-filtered in a strict manner, and this is followed by information analysis of these high-quality clean reads. Compared to the cattle reference genome, as assessed by TopHat (v2.0.9) software, a total of 15,557 genes (14,935 known genes and 622 new genes) and 15,574 genes (14,953 known genes and 621 new genes) were identified from the BVDV-infected MDBK group and mock-infected MDBK group, respectively. Subsequently, compared to the mock-infected MDBK group, the DEGs in the BVDV-infected MDBK group were screened according to the parameters: false discovery rate (FDR) < 0.05 and |log2FC| > 1. As presented in **Figure 2A**, a total of 665 DEGs were obtained from the BVDV-infected MDBK group, including 274 significantly up-regulated genes and 391 significantly down-regulated genes. Additionally, a volcano plot for the DEG distribution was generated (**Figure 2B**).

**Figure 3:**
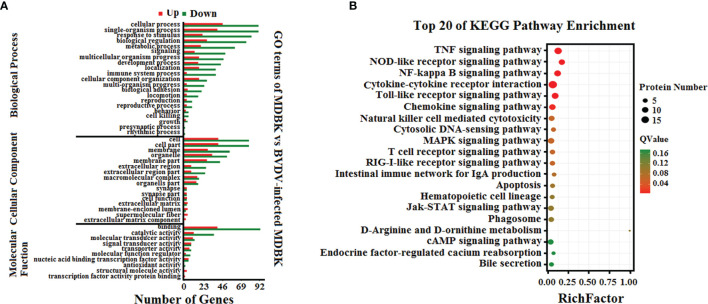
**(A)** GO annotation and **(B)** KEGG pathway enrichment analysis of the DEGs in the RNA-seq transcriptomics data of BVDV-infected MDBK cells compared to mock-infected MDBK cells.

To analyze the biological functions of the DEGs, GO annotation and KEGG pathway enrichment analyses were performed. As presented in **Figure 3A**, GO terms in the biological process category of most DEGs were primarily involved in single-organism, metabolic, or cellular processes, response to stimulus, and biological regulation. It was noteworthy that a number of DEGs were involved in cell killing, immune system process, and presynaptic process. In the cell component category, a large number of DEGs were involved in cell, cell part, membrane, and organelle. Notably, certain DEGs were involved in extracellular matrix, membrane-enclosed lumen, and supramolecular fiber. In the molecular function category, the GO terms of the DEGs were primarily involved in binding and catalytic activity. Significantly, we also determined that the down-regulated DEGs in the BVDV-infected MDBK cells were primarily involved in immune system process, cell killing, and response to stimulus, while the up-regulated DEGs were predominantly involved in cell part, membrane part, binding, transcription factor activity protein binding, and cell process. This indicates that BVDV may evade host innate immunity by inhibiting the host immune system and host apoptosis at the early stage of infection and may also utilize cellular components to promote virus replication. Thus, further experimental studies are required to explore these mechanisms. Subsequently, the results of KEGG pathway enrichment analysis of the DEGs revealed that the DEGs were primarily associated with cytokines and cytokine receptor interactions, the NOD-like receptor signaling pathway, TNF signaling pathway, Toll-like signaling pathway, JAK-STAT signaling pathway, MAPK signaling pathway, NF-κB signaling pathway, RIG-I-like receptor signaling pathway, and apoptosis (**Figure 3B**). Among these, the RIG-I-like receptor signaling pathway, Toll-like signaling pathway, and JAK-STAT signaling pathway play an important role in host innate immunity, and the TNF signaling pathway and NOD-like receptor signaling pathway play an important role in regulating host immunity. Our data provide important background information to explore BVDV-host interactions at the mRNA level.

### Quality Evaluation of Proteome and Different Proteins Analysis

**Figure 4:**
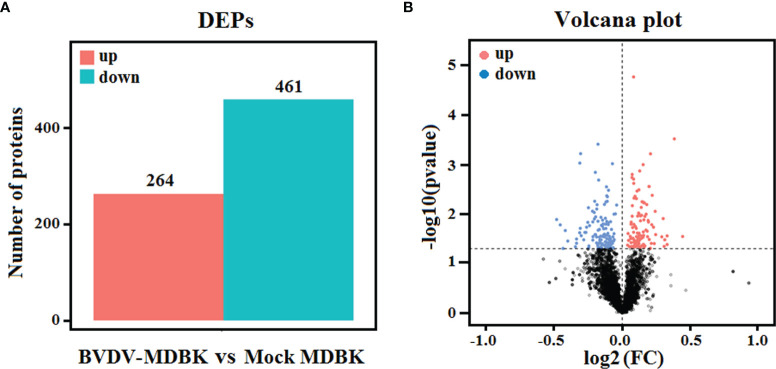
**(A)** Statistics for the DEPs in iTRAQ-based proteomics data for BVDV-infected MDBK cells compared to mock-infected MDBK cells. The red histogram represents the number of up-regulated DEPs, and the blue histogram represents the number of down-regulated DEPs. **(B)** Volcano plot of the DEPs. The red dots represent significantly up-regulated proteins, blue dots represent significantly down-regulated proteins, and black dots represent insignificantly DEPs.

Using mock-infected MDBK cells as a negative control, proteome sequencing for the BVDV-infected MDBK cells was performed using iTRAQ followed by LC-MS/MS analysis. Compared to the mock-infected MDBK group, proteins with *P*<0.05 were considered to be differentially expressed proteins (DEP). As presented in **Figure 4A**, a total of 725 DEPs were identified, including 264 up-regulated DEPs and 461 down-regulated DEPs. Meanwhile, a volcano plot for the DEPs distribution was generated (**Figure 4B**).

**Figure 5:**
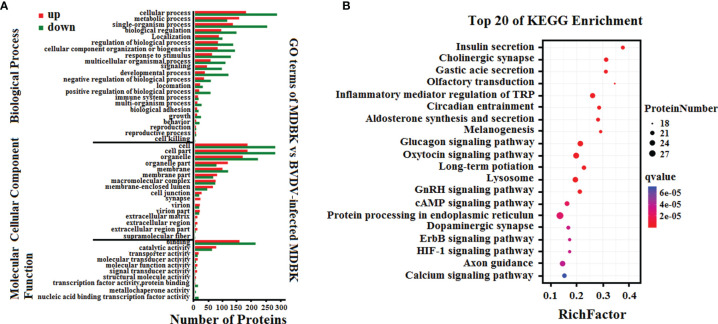
**(A)** GO annotation and **(B)** KEGG pathway enrichment analysis of the DEPs in the iTRAQ-based proteomics data for BVDV-infected MDBK cells compared to mock-infected MDBK cells.

Subsequently, GO annotation and KEGG pathway enrichment analysis of these DEPs was performed. As presented in **Figure 5A**, GO annotation in the biological process category of the DEPs revealed that majority of the DEPs were primarily involved in cell, metabolic, and single-organism process, biological regulation, and regulation of biological process. Noticeably, several DEPs were involved in negative regulation of biological process, immune system process, response to stimulus, and reproduction. In the cell component category, many DEPs were involved in cell, cell part, organelle, and organelle part, and few DEPs were involved in cell junction, virion, and extracellular region. In the molecular function category, the GO terms for the DEPs were predominantly involved in binding, and catalytic activity. Moreover, we observed that the number of down-regulated DEPs was higher compared to that of up-regulated DEPs for most GO terms, particularly the GO terms related to host innate immunity. This indicated that BVDV could inhibit the host immune system at the early stage of viral infection to facilitate its survival. However, in the metabolic process term, the number of up-regulated DEPs was significantly higher compared to that of down-regulated DEPs, indicating that BVDV could utilize the host metabolism to facilitate viral replication. This was consistent with the result of the transcriptome analysis. Further, the KEGG pathway enrichment analysis results revealed that the DEPs were primarily associated with protein processing in endoplasmic reticulum, oxytocin signaling pathway, glucagon signaling pathway, lysosome, and inflammatory mediator regulation of TRP channels (**Figure 5B**). Interestingly, several signaling pathways were associated with host metabolism, particularly lipid metabolism and sugar metabolism. Moreover, a small number of DEPs were associated with melanogenesis, the ErbB signaling pathway, and the HIF-1 signaling pathway, and these were primarily involved in cell proliferation. These data provide important background information to elucidate BVDV-host interactions at the protein level.

### Combined Analysis of Transcriptome and Proteome Data

**Figure 6:**
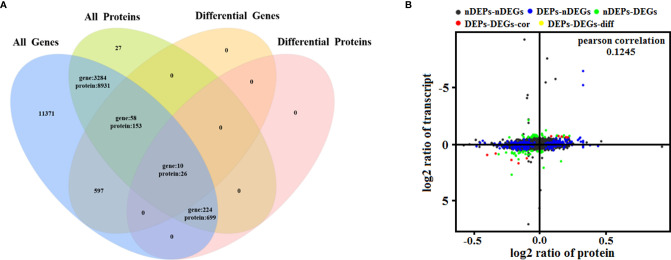
Quantitative analysis of transcriptome proteome associations. **(A)** Venn diagram of all mRNAs and proteins. ‘All genes’ represents the detected genes, and ‘All proteins’ represents the detected proteins. **(B)** In the four quadrant diagram, the differential genes and differential proteins were screened according to the threshold of transcriptome and proteome. The black dots represent non-DEPs and non-DEGs, the green dots represent DEGs but non-DEPs, the blue dots represent DEPs but non-DEGs, the red dots represent DEGs and DEPs with the same trend, and the yellow dots represent DEGs and DEPs with the opposite same trend.

**Figure 7:**
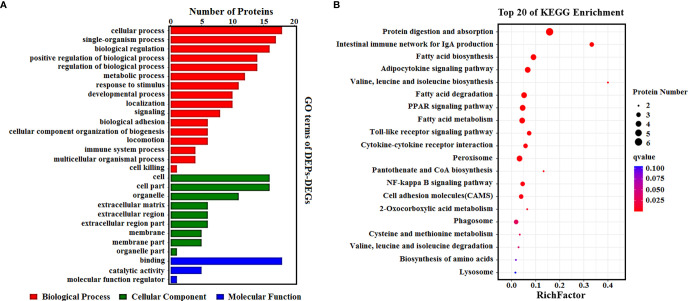
**(A)** GO enrichment analysis for DEPs_DEGs. Different colors represent different types of GO terms; **(B)** KEGG pathway analysis for DEPs_DEGs. The top 20 significant enrichment pathways were listed.

**Figure 8:**
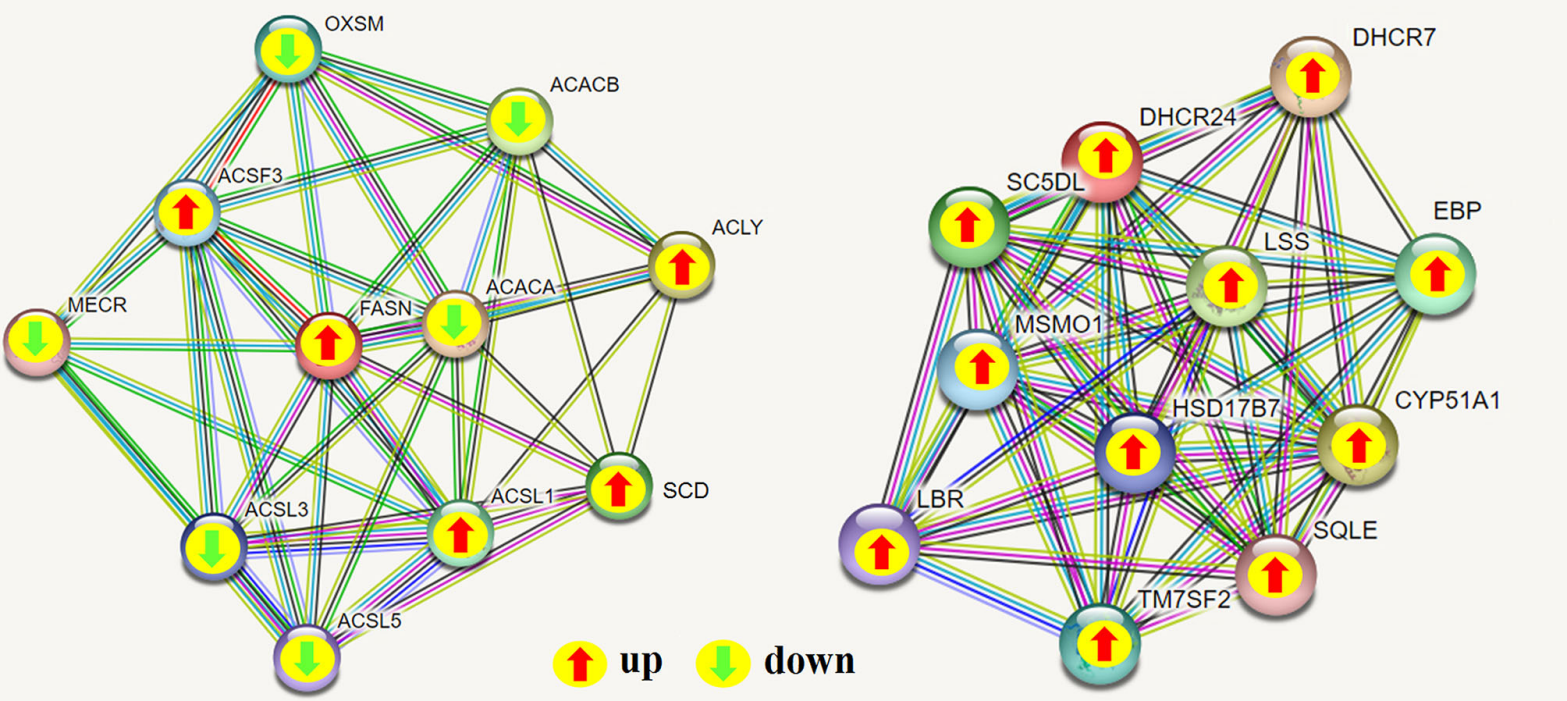
Protein-protein interaction (PPI) network analyses of DEPs involved in regulating lipid metabolism were performed using the STRING database while focusing on FASN and DHCR24. The green arrow represents down-regulated expression, and the red arrow represents up-regulated expression; green line: gene neighborhood; red line: gene fusion; blue line: gene co-occurrence; yellow line: text mining; black line: co-expression; light purple line: protein homology.

To determine the complementarity and integration of mRNAs and proteins, we analyzed the correlation between the DEGs and DEPs. As presented in **Figure 6A**, there was a large amount of coverage for proteins that were encoded by abundant transcripts. Among these, a total of 665 DEGs and 725 DEPs were displayed, including ten genes that were differentially expressed both at mRNA and protein levels (**Table 2**). Moreover, to investigate if the variations in protein levels were correlated with changes in the corresponding transcripts in the BVDV-infected MDBK group compared to those in the mock-infected MDBK group, the distribution of the corresponding ratios of mRNA to protein was analyzed. As presented in **Figure 6B**, almost all ratios of mRNA to protein were concentrated at the center of the plots, and only some gene and protein expression levels were inconsistent. GO annotation and KEGG pathway enrichment analysis were performed for mRNAs and proteins exhibiting the same expression trends. GO analysis revealed that these genes primarily belonged to cellular process, single-organism process, biological regulation, cell, cell component, organelle, binding, and catalytic activity (**Figure 7A**), and they were primarily involved in metabolism, protein digestion and absorption, fatty acid biosynthesis, cytokine-cytokine receptor interaction, and the PPAR signaling pathway as analyzed by KEGG pathway enrichment (**Figure 7B**). Additionally, we analyzed the regulation networks of lipid metabolism pathway focusing on FASN and DHCR24, and the results of the protein-protein interaction (PPI) that was elucidated using the STRING (https://string-db.org/) database revealed that DHCR24 was highly interconnected with relevant proteins involved in cholesterol synthesis such as DHCR7, MOMS1, and LSS; and that FASN was highly interconnected with relevant proteins involved in fatty acid biosynthesis such as ACACA, OXSM, and ACSL1 (**Figure 8**).

**Table 2:** Ten genes were significantly differentially expressed both in mRNA and proteins levels.

Genes	Gene ID	Protein ID
ACSL	ncbi_537161	NM_001076085.1
LGALS9	ncbi_510813	XM_024979912.1
BCAT2	ncbi_281643	NM_001013593.2
CTSC	ncbi_352958	NM_001033617.2
CD40	ncbi_286849	NM_001105611.2
COL18A1	ncbi_508076	XM_024975114.1
CTSV	ncbi_281108	NM_174032.2
MRC2	ncbi_529049	NM_001192670.1
NT5E	ncbi_281363	NM_174129.3
PLAUR	ncbi_281983	NM_174423.3

### Verification of DEGs by RT-qPCR and of DEPs by Western Blot

**Figure 9:**
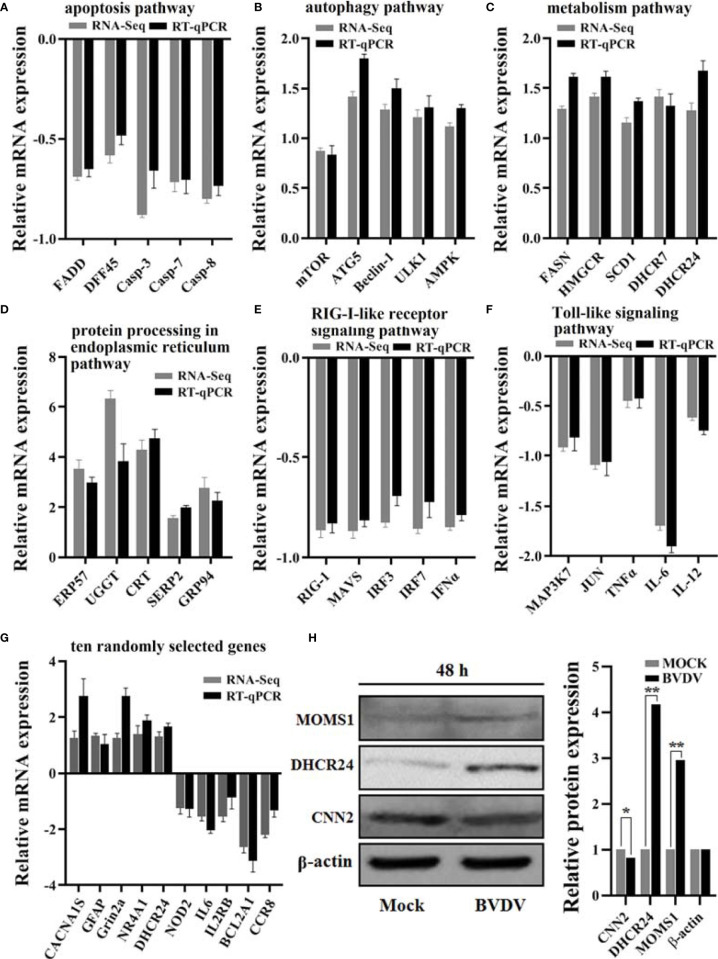
Verification of the RNA-seq-based transcriptomics data and the iTRAQ-based proteomics data obtained in this study. The DEGs from RNA-seq-based transcriptomics data analysis that primarily involved in **(A)** the apoptosis pathway, **(B)** the autophagy pathway, **(C)** the metabolism pathway, **(D)** the protein processing in endoplasmic reticulum pathway, **(E)** the RIG-I-like receptor signaling pathway, and **(F)** the Toll-like signaling pathway were verified using RT-qPCR and **(G)** the 10 randomly selected genes. Additionally, **(H)** the protein expression levels of the DEPs DHCR24, MOMS1, and CNN2 were verified using western blot. *P-value < 0.05; **P-value < 0.01.

RT-qPCR assay was performed to verify the effectiveness of RNA-seq-based transcriptome sequencing data. The relative expression levels of all genes selected from the transcriptome sequencing data exhibited similar trends when compared to the RNA-seq results (**Figures 9A–G**). Additionally, the DEPs MOMS1, DHCR24, and CNN2 were selected for evaluating the iTRAQ results using western blot analysis, and our results revealed that the expression levels of MOMS1 and DHCR24 were up-regulated and that of CNN2 was down-regulated in BVDV-infected MDBK cells compared to the expression levels in the mock-infected MDBK cells (**Figure 9H**). This was consistent with the iTRAQ-based proteomic analysis data.

## Discussion

BVD-MD caused by BVDV is an important infectious disease in cattle that has resulted in significant economic losses to the cattle industry worldwide. BVDV has evolved strategies such as promoting massive apoptosis of monocytes and inhibiting interferon antiviral response to evade host antiviral innate immunity to promote virus survival. Although a number of underlying mechanisms of BVDV-host interactions have been elaborated clearly, the detailed mechanisms involved in viral replication, pathogenesis, and host innate immunity evasion remain to be elucidated. In this study, in order to further explore BVDV-host interactions, particularly how BVDV regulates the host to promote viral replication, the one-step growth curve of BVDV strain was determined firstly, and we can see that the viral titer increased faster at 48 h than at other time points post BVDV infection. Therefore, we collected the cell samples at 48 h post BVD infection for the integrated analysis of RNA-seq-based transcriptomics profiles and iTRAQ-based proteomics profiles of the BVDV-infected host cells. Although there has transcriptomic sequencing analysis for BVDV-infected host cells at 2 h, 6 h, 12 h, and 24 h post BVDV infection (21) and proteomic analysis for BVDV-infected host cells at 12 h, 24 h, and 48 h post BVDV infection (22), and a lot of DEGs and DEPs were obtained, just only one omics data type analysis is limited to correlation between mRNA and protein abundances in BVDV-infected host cells.

Innate immunity plays an important role in the host resistance to virus infection and invasion. Typically, viral nucleic acids and proteins act as important pathogen-associated molecular patterns (PAMSs) that can be recognized by pattern-recognition receptors (PRRs) to facilitate the activation of several pathways, including the Toll-like signaling pathway, and the RIG-I signaling pathway, resulting in a large increase in inflammatory cytokines and the secretion of chemokines (27, 28). However, genes related to innate immunity such as IFNα, TNF, KKR, IL-6, IL-12, and IL-1β were down-regulated in the transcriptomic profiles of BVDV-infected MDBK cells. Type-1 IFNs are important host antiviral cytokines (29). Currently, the molecular mechanisms by which BVDV inhibits interferon production are not fully understood. Certain studies have demonstrated that the BVDV Npro protein can inhibit the activation of the type-1 IFN by degrading the transcription factor IFN regulatory factor 3 (IRF3) (30, 31). Moreover, PKR, OAS, Mx1, and ISGs are important host antiviral proteins that can be induced by IFN (32–35). According to our RNA-seq data analysis, the expression levels of these antiviral proteins were down regulated, and this was consistent with the results of previous studies, thus indicating that BVDV can down-regulate the expression level of host antiviral proteins during the early stage of virus infection. Hepatitis C virus (HCV), like BVDV, also belongs to the genus *Pestivirus* of the *Flaviviridae*, and researchers have determined that at the early stage of HCV replication, inflammatory factors can induce cellular immunity to eliminate the virus and further promote the release of inflammatory mediators (36). However, BVDV could infect bovine monocytes and further inhibit the expression of pro-inflammatory cytokines such as TNF-α, IL-1β, and IL-6 (37), which was consistent with our RNA-seq analysis data. Typically, the NLRP3 inflammasome could be activated by PAMPs and danger-associated molecular patterns (DAMPs) to further promote the maturation and release of IL-1β and IL-18 to mediate antiviral responses (38, 39). In this study, we observed that although the NLRP3 inflammasome was up-regulated in BVDV-infected MDBK cells, the expression of inflammatory factors (IL-1β, IL-6, IL-12, and particularly IL-1β) was inhibited. Combined with the results indicating that the expressions of antiviral proteins were also inhibited, we speculated that BVDV would have to create a favorable environment for facilitating its survival by regulating host innate immunity through mechanisms affecting inhibition of inflammatory factors production and IFN production while the host simultaneously initiates inflammatory signaling pathways to resist viral infection. However, the underlying specific mechanisms must be further explored.

A previous study reported that classical swine fever virus (CSFV) could induce lymphocyte apoptosis during infection (40). Recently, researchers have confirmed that BVDV could promote the expression of PD-1 to induce peripheral blood lymphocyte apoptosis (41). In this study, we observed that certain anti-apoptotic proteins such as BCL2A1, BIRC3, and NGF were significantly down-regulated in the BVDV-infected MDBK cells compared to their levels in the mock-infected MDBK cells. Of these proteins, NGF could promote cell survival by combining with TrkA (42), while BIRC3 could inhibit caspase-mediated cell apoptosis (43). Moreover, the expression level of the key apoptotic regulatory protein caspase 3 was also significantly down-regulated in MDBK cells after BVDV infection. These data imply that BVDV infection induced apoptosis. Interestingly, the expression level of another important anti-apoptotic protein (Bcl-2) was significantly up-regulated in the MDBK cells after BVDV infection. The existing research data has confirmed that the anti-apoptotic Bcl-2 protein resides on the outer mitochondrial membrane and can suppress apoptosis by inhibiting the activation of the pro-apoptotic molecules Bax and Bak (44) and can also affect apoptosis induced by the Epstein-Barr virus (45). To explain our results, we speculated that cell-autonomous apoptosis for inhibiting virus replication was simultaneously present with viral anti-apoptosis for facilitating its replication at the early stage of BVDV infection; however, the precise regulatory mechanisms must be further elucidated. Additionally, it remains unclear if non-coding RNAs (lncRNAs/microRNAs) were also involved during the process of BVDV-host interactions, and the exact mechanisms have to be further investigated.

The endoplasmic reticulum (ER) plays an important role in regulating protein synthesis and cellular metabolism (46). Meanwhile, ER stress can be induced by virus infection, and this can further induce both pro- and anti-apoptotic signaling (47). In this study, many DEPs that were enriched in protein processing in the endoplasmic reticulum signaling pathway were up-regulated, including ERP25, GPR78, GRP94, CRT, UGGT, and NEF. Of these, GPR78 and GPR94 are critical ER-resident molecular chaperones that promote protein folding. The envelope glycoproteins of HCV can activate the GPR78 and GPR94 promoters (48). Based on this, we concluded that BVDV infection can activate GPR78 and GPR94. Normally, ER transmembrane kinase (PERK) is maintained in an inactive, monomeric state by association with the GRP78 and GRP94 that are released, upon ER stress, from PERK to assist in protein folding (49, 50). Thus, we speculate that BVDV-induced ER stress is caused by the presence of unfolded or unassembled viral proteins. A previous study reported that BVDV-induced ER stress could initiate an apoptotic cascade involved in down-regulation of Bcl-2 expression (50). However, the molecular relationships between the BVDV-induced ER stress signal and activation of downstream pathways involved in apoptosis require further study.

Viruses are obligate intracellular parasites that depend upon the host cell for raw materials and energy necessary for all biological processes, particularly nucleic acid synthesis, and protein synthesis, processing, and transport (51–55). Many viruses can alter the host metabolic network to create a suitable intracellular microenvironment for their life cycles. For example, ZIKV infection altered host glucose metabolism in HFF1 cells (56), and adenovirus infection promoted host cell anabolic glucose metabolism and lactate production (57). Currently, an increasing number of studies have demonstrated that there is a close relationship between cholesterol metabolism and innate immunity. Innate immune signals can regulate the transport, storage, and release of cholesterol (58). The steroid pathway is the key pathway related to lipid storage and metabolism, which can regulate glucose metabolism, cholesterol metabolism, water salt metabolism, bile acids, and bile alcohol synthesis and degradation (59, 60). BVDV is often used as an alternative virus model for hepatitis C virus (HCV) research. HCV is a lipophilic virus, and its replication and infectivity is regulated by the lipid state of cells (61). HCV can utilize the LDL receptor to enter cells and form replication complexes on the lipid rafts (62). During the process of HCV replication in host cells, lipid droplets on the endoplasmic reticulum can be utilized to form virus particles, its release to the extracellular environment also requires the assistance of very low density lipoproteins (VLDLs) (63, 64), indicating that lipid formation is highly essential for HCV propagation. In this study, we revealed that the up-regulated DEPs SREBP, ERG25, HMGCR, MOMS1, DHCR7, DHCR24, SC4MOl, FAH, OLR1, and MMP-1 are primarily enriched in the steroid synthesis pathway. Of these, the SREBP (sterol regulatory element binding protein) transcription factor is a major regulator of lipid homeostasis that can activate 3-hydroxy-3-methyl-glutaryl coenzyme A reductase (HMGCR), which in turn can promote cholesterol synthesis (65). Metabolic enzymes, such as DHCR7 and DHCR24, involved in the cholesterol synthesis pathway play an important role in virus replication (66, 67), and a synergistic relationship exists between SREBP and DHCR24 (68, 69). Therefore, BVDV can utilize the host lipid substances to promote viral replication; however, the underlying molecular mechanisms require further investigation. Moreover, how BVDV utilizes sugar metabolism to promote its replication, and how BVDV utilizes sugar metabolites, particularly lactate, to inhibit the host innate immunity remains unclear. These provide interesting directions for future research studies.

Additionally, cell autophagy plays an important role in clearing intracellular substances and maintaining the stability of the intracellular environment (70), and this can also affect the life cycle of the virus by regulating cell metabolism. For example, NDV infection has been demonstrated to regulate host glucose metabolism through mitochondrial autophagy (71). Typically, lipids can be used as autophagy substrates. For example, macrophages can use lysosomal degradation pathway to consume intracellular lipid droplets (72, 73). Occasionally, host cells can regulate cellular energy and lipid storage by phagocytizing lipids under specific conditions (74). A previous study demonstrated that the loss of the lipid droplet protein AUP1 can cause defective virus production in DENV-infected cells, and this could be rescued when AUP1 was reconstituted with a wild type copy (75). In this study, our data revealed that the expression levels of LC3 and Beclin1 that are involved in the autophagy pathway were significantly up-regulated. Thus, combined with the lipid metabolic pathway analysis described above, we speculate that BVDV infection could trigger lipophagy to further drive virus production; however, this requires experimental validation.

**Figure 10:**
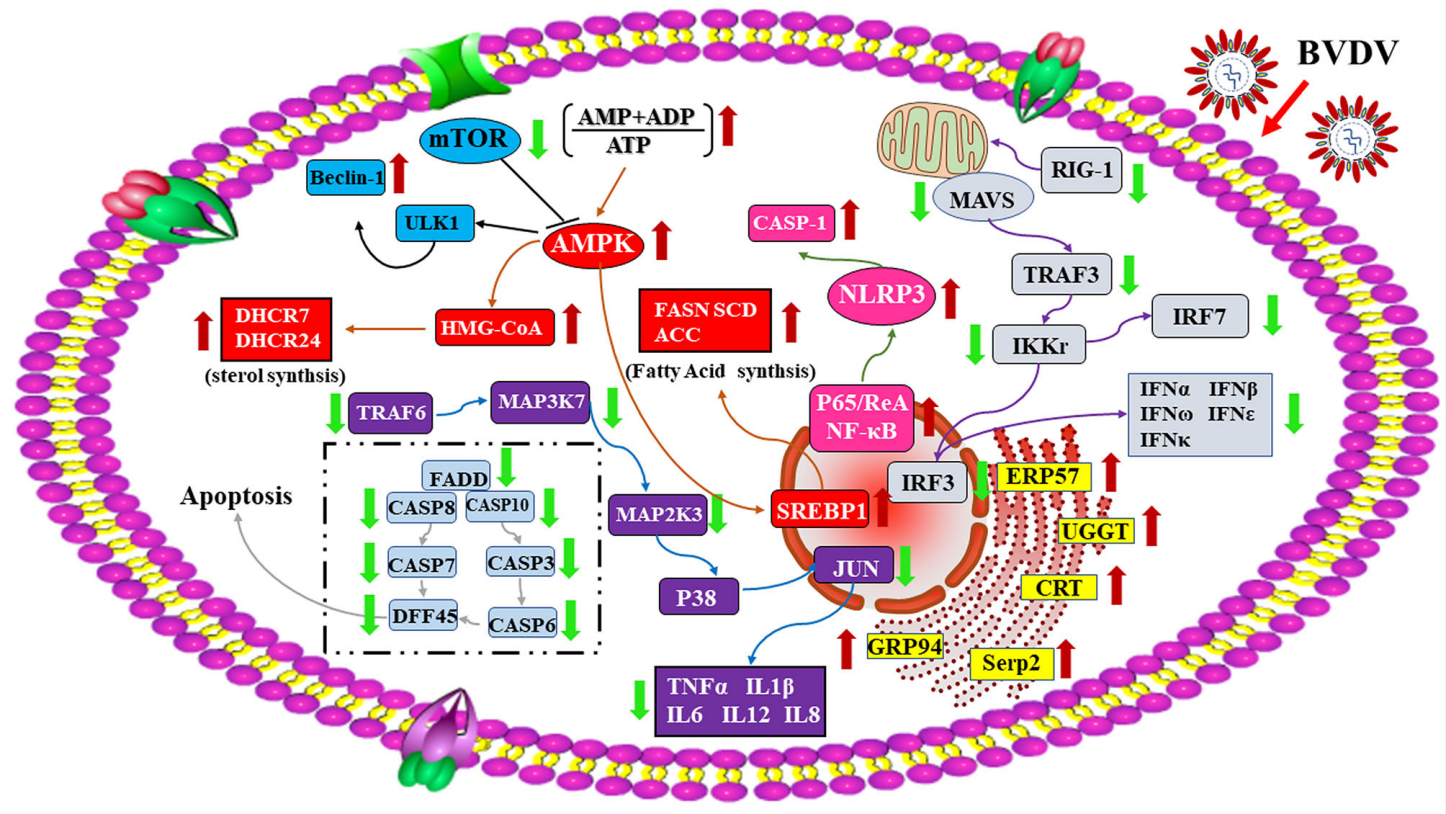
A global overview of the major signaling pathway involved in BVDV-MDBK cell interactions. The red arrow represents up-regulated DEPs, and the green arrow represents down-regulated DEPs.

In conclusion, the RNA-seq-based transcriptomics profiles and the iTRAQ-based proteomics profiles of BVDV-infected MDBK cells that were obtained in this study in combination with the significant DEGs and DEPs that were identified, compared to mock-infected MDBK cells (**Figure 10**), provide a deeper insight into BVDV-host interactions during BVDV infection and an important basis to further elucidate the underlying mechanisms involved in viral replication, pathogenesis, and evasion of host innate immunity in the context of BVDV.

## Data Availability Statement

The datasets presented in this study can be found in online repositories. The names of the repository/repositories and accession number(s) can be found below: https://www.ncbi.nlm.nih.gov/, accession ID: PRJNA596327.

## Author Contributions

Conceptualization, YX and XG. Methodology, YM, LW, and XH. Investigation, YM, XY, KZ, and YY. Data curation, YM, YW, XJ, and XS. Writing—original draft preparation, YM. Writing—review and editing, YX. Supervision, XG. Project administration, LW and YX. Funding acquisition, YX. All authors read and approved the manuscript.

## Funding

This work was supported by the Research and Development Fund of Zhejiang A&F University (grant number 2021FR034) and the Heilongjiang Provincial Natural Science Foundation of China (grant number LH2021C046).

## Conflict of Interest

The authors declare that the research was conducted in the absence of any commercial or financial relationships that could be construed as a potential conflict of interest.

The reviewer XQ declared a shared affiliation with the authors YM, LW, XJ, XY, XH, KZ, YY, XS, and XG to the handling editor at the time of review.

## Publisher’s Note

All claims expressed in this article are solely those of the authors and do not necessarily represent those of their affiliated organizations, or those of the publisher, the editors and the reviewers. Any product that may be evaluated in this article, or claim that may be made by its manufacturer, is not guaranteed or endorsed by the publisher.
